# Effect of automated drug distribution systems on medication error rates in a short-stay geriatric unit

**DOI:** 10.1111/jep.12202

**Published:** 2014-06-11

**Authors:** Etienne Cousein, Julie Mareville, Alexandre Lerooy, Antoine Caillau, Julien Labreuche, Delphine Dambre, Pascal Odou, Jean-Paul Bonte, François Puisieux, Bertrand Decaudin, Patrick Coupé

**Affiliations:** 1 Department of Biopharmacy, Galenic and Hospital Pharmacy, EA GRIIOT, UDSL, Université Lille Nord de FranceLille, France; 2 Department of Biopharmacy, Galenic and Hospital Pharmacy, EA GRIIOT, UDSL, Université Lille Nord de FranceLille, France; 3 Department of Pharmacy, Valenciennes General HospitalValenciennes, France; 4 Geriatric Short Stay Unit, Valenciennes General HospitalValenciennes, France; 5 Department of Biostatistics, UDSL, Université Lille Nord de FranceLille, France; 6 Department of Pharmacy, University Hospital of LilleCHU Lille, Lille, France; 7 Department of Analytical Chemistry, EA GRIIOT, UDSL, Université Lille Nord de FranceLille, France; 8 Geriatric Department, University Hospital of LilleLille, France; 9 Laboratory for the Biology of Vascular Aging, UFR Médecine, Université Lille Nord de FranceLille, France

**Keywords:** clinical audit, drug distribution system, elderly, medical order entry systems, medication errors, robotics

## Abstract

**Rationale, aims and objectives:**

To assess the impact of an automated drug distribution system on medication errors (MEs).

**Methods:**

Before-after observational study in a 40-bed short stay geriatric unit within a 1800 bed general hospital in Valenciennes, France. Researchers attended nurse medication administration rounds and compared administered to prescribed drugs, before and after the drug distribution system changed from a ward stock system (WSS) to a unit dose dispensing system (UDDS), integrating a unit dose dispensing robot and automated medication dispensing cabinet (AMDC).

**Results:**

A total of 615 opportunities of errors (OEs) were observed among 148 patients treated during the WSS period, and 783 OEs were observed among 166 patients treated during the UDDS period. ME [medication administration error (MAE)] rates were calculated and compared between the two periods. Secondary measures included type of errors, seriousness of errors and risk reduction for the patients. The implementation of an automated drug dispensing system resulted in a 53% reduction in MAEs. All error types were reduced in the UDDS period compared with the WSS period (*P* < 0.001). Wrong dose and wrong drug errors were reduced by 79.1% (2.4% versus 0.5%, *P* = 0.005) and 93.7% (1.9% versus 0.01%, *P* = 0.009), respectively.

**Conclusion:**

An automated UDDS combining a unit dose dispensing robot and AMDCs could reduce discrepancies between ordered and administered drugs, thus improving medication safety among the elderly.

## Introduction

In December 1999, the Institute of Medicine reported that medical errors were associated with up to 98 000 deaths and more than 1 million injuries each year in the United States [1]. Medication errors (MEs) alone, occurring either inside or outside the hospital, were estimated to account for more than 7000 deaths in 1996 [2]. A study of 10 North Carolina hospitals conducted between January 2002 and December 2007 was consistent with persistent safety concerns, reporting 56.5 incidents per 1000 patient-days of which 27% were related to medications [3]. An estimate of 63.1% of these were deemed to be preventable. Among inpatients, medication management of older people is of special importance because of their sensitivity to the adverse effects of drugs. Given the frequent coexistence of a large number of pathologies, and considering that surgical procedures or recommendations about life patterns are often inappropriate, combinations of drug therapies are sometimes necessary. Organic weaknesses also require sharp adjustments to these therapies. Admission itself to a short-stay geriatric unit may in fact be directly linked to medication management. During the stay, any discrepancy between the drugs prescribed by the physician and those administered by the nurse may compromise medication management, so lengthening the stay, or worse, harming the patient.

MEs are a well-known issue in hospitals, but are still difficult to address because of the large number of health care professionals involved in patient medication management with several tasks to be completed in prescribing, dispensing and administering medications to one patient. Some interventions have been shown to reduce errors, such as implementing computerized provider order entry (CPOE) systems 4–6, or bar code scanning at the point of care [7]. To improve drug distribution, one of the commonly proposed solutions is to switch from a ward stock system (WSS), where nurses order drugs in bulk from the pharmacy, to a daily unit dose dispensing system (UDDS), where pharmacy staff prepares for each patient the drugs required for a 24-hour period, sorted by administration time, according to physicians' orders. The goal of these interventions is to ensure appropriate medication administration and to reduce costs. Even though quite common in North America, automated drug dispensing systems are still rarely encountered in Europe, while concern about inpatient medication management safety is rising. In view of the investments required along with considerable organizational changes, health professionals are eager for some efficiency data, adapted to their own country and work settings.

Some studies have investigated the impact of a UDDS, but only a few have used an observation-based method, making interpretation difficult 8–10. Some studies have focused on automated medication dispensing cabinets (AMDCs) [11,12] the results of which suggest that these systems could reduce MEs. However, to our knowledge, an observation-based assessment of a medication dispensing system consisting of a UDDS and an AMDC has not yet been made. The aim of this observational before-and-after study is to assess the efficacy of a daily UDDS on discrepancies between what is prescribed and what is administered to the patient.

## Methods

Taking into account the observational setting of the study, a waiver of consent was obtained from the institutional review board of our hospital.

### Setting

This study was carried out in a 40-bed short-stay geriatric ward in a 1800-bed French general hospital. Medication administration errors (MAEs) were estimated from an observation-based study [13] from 14 June to 9 September 2010 for the WSS period; from 20 January to 13 March 2012 for the UDDS1 period; and from 14 March to 19 April 2012 for the UDDS2 period.

### Definitions

Definitions were based on those of Allan and Barker [14]. A MAE was defined as a dose of medication administered to a patient that deviated from the doctor's order. MAEs were classified as wrong dose, wrong drug, wrong time of administration, and omission. Expired drugs could not be assessed as medications were mostly out of their original packaging during the WSS period. Wrong dosage forms were included in the wrong drug category. The denominator used to calculate MAE rates was the total number of opportunities for errors (OEs). OE was defined as any dose administered or any dose prescribed but omitted.

### Procedure

During the WSS period, prescriptions on paper were placed in a medical record and then transcribed by nurses onto a drug administration sheet. Drugs were prepared every day by nurses using a large floor stock of formulary drugs, which were ordered from the pharmacy twice a week by nursing staff without regard to physicians' orders. Daily management of the floor stock is the responsibility of the nursing staff. The pharmacy reviews this stock twice a year. For non-formulary drugs, and formulary drugs unavailable on the floor stock level, the pharmacy delivers the medications based on a prescription faxed to the pharmacy and checked by a pharmacist. Orders are only applicable to medications unavailable on the floor stock level. The pharmacy is open from Monday to Saturday from 0800 to 1830 h, with a resident pharmacist available on call at other times.

During the UDDS period, physicians' orders placed during morning and afternoon rounds were addressed by a unit dose-delivering robot (Pillpick Pharmacy Automation System, Swisslog, Maranello, Italy), which prepares daily therapies in bags attached with a ring and sorted according to administration time. Each bag contains one dose (tablet, capsule, vial, half/quarter-tablet, sachet, unit dose collyrium) to be delivered to the patient, and indicates drug name and dosage, batch, and expiration date. Most drugs are packaged for single-dose dispensing. A label placed on each ring indicates the patient's name, ward, bed and all the drugs contained in the bag. Medications that cannot be handled by the robot (cold storage drugs, bulky bottles, intravenous delivery bags) are dispensed by racks or automated storage systems (Rotomat, Hanel, Bad Friedrichshall, Germany) for each patient with a label printed via the pharmacy Warehouse Management Software (WMS) (Copilote, Savart & Michel, Meylan, France), indicating the patient's name, unit, room, dosage and time of administration. Pharmacy technicians check the rings, label the medications not handled by the robot, and fill the appropriately assigned drawers on the nurses' medication carts. Each drawer contains the drugs required for a given patient's scheduled medication for the 24 hours to come. When an order is entered into the CPOE outwith the operating hours of the robot, drugs are available for the nurses via an AMDC (Pyxis MedStation 3500, CareFusion, San Diego, CA, USA), connected to the pharmacy WMS and to the admission/discharge/transfer software. After signing on to the AMDC, nurses can select the patient and the drugs to be administered to that patient, as physicians' orders are displayed for each patient on the AMDC. The pharmacy staff are responsible for AMDC restocking and inventory. If a drug is not available in the AMDC, nursing staff can reach the pharmacy from Monday to Saturday from 0800 to 1830 h, or via a resident pharmacist available on call at other times.

UDDS has two variants, with or without electronic medication administration record (eMAR), which we have classified as UDDS1 and UDDS2.

UDDS1: computerized physician order entry (CPOE) without eMAR
  Orders are placed in a CPOE system based on the pharmacy WMS, and then printed simultaneously in the ward and in the pharmacy. Copies are used by both pharmacists and nurses to check the orders and record medication administration. No decision support system exists at any stage in the drug delivering system. If any drug-related problem is identified by the pharmacist, it is discussed in the ward with the physicians or by phone if face-to-face discussion is not possible.
UDDS2: computerized physician order entry (CPOE) including eMAR (UDDS2)

Orders are placed in an electronic patient record, including CPOE, pharmacy orders monitor and eMAR (Millennium, Cerner Corporation, Kansas City, MO, USA). Pharmacist interventions are entered into the patient record and discussed in the ward with the physicians by phone if face-to-face discussion is not possible.

### Measurements

Researchers (EC, JM, VK, pharmacists and OD, quality engineer) accompanied drug administration rounds in a randomly selected ward section, noting on a standardized form their duration and every oral-route drug administered to each patient. The first round observed by each researcher was debriefed by the research group. Oral-route drugs were usually scheduled to coincide with the three medication administration rounds (morning, noon, evening). Some drugs administered outwith these rounds were not included in the study. The colour and shape of drugs that were no longer in their original packaging were noted and then compared to packaged drugs. Administrations and prescriptions were compared so that MAEs could be assessed. Observers were instructed to stop the nurse if any harmful error seemed likely to happen.

The duration of nurses' rounds was noted and included in the study from the first medication administered to the first patient in the section to the last medication administered to the last patient in the section. This implies that the duration includes not only medication administration but also (for example) administration records, discussion with the patient or family, and phone calls to physicians. Other factors that could possibly have an impact on medication administration safety, such as the mean number of medications per patient, were also noted.

Four physicians (CJG, GG, AL, AC) from the short-stay geriatric unit who were not involved in the data collection retrospectively reviewed the errors, blinded to the WSS and UDDS study periods. Physicians classified errors into the following categories: no harm, minimum harm without monitoring expected, monitoring, and need for therapy or intervention.

### Data analysis

Calculations were made of the overall rate of MAEs and the percentage of patients experiencing ≥1 MAE in each study period. For both of these, the 95% confidence interval (CI) was established as well as the absolute and relative risk difference between the two study periods (WSS versus UDDS period). The number of patients requiring treatment to prevent MAE was also calculated as the inverse of absolute risk reduction [15].

Comparison of patients, rounds and other collected data for the two periods was made using the Student's *t*-test for quantitative variables and the chi-square test (or Fisher's exact when expected cell frequency was <5) for qualitative variables. All comparisons performed on the OE as statistical unit were made using a multilevel regression model so as to take into account the clustering of OEs on patients (level 1) and nurses (level 2). We used a random-intercept logistic regression model including period as fixed effect to estimate (using the least-square mean of the logit) and compare MAE rates.

Previous studies [9,16] put forward a 12% MAE rate estimation for WSS, and a relative risk reduction of 40%. For a power of 80%, with a two-sided alpha level of 5%, we calculated that at least 587 OEs would be required in each group.

Statistical testing was performed at the two-tailed α level of 0.05. Data were analysed using the SAS software package, release 9.3 (SAS Institute).

## Results

### Descriptive data

Table 1 shows the patient and unit characteristics during the WSS and UDDS periods. The characteristics of the rounds observed are presented in Table 2.

**Table 1:** Patient and unit characteristics for each period

Period	WSS	UDDS	*P*
*Patient characteristics*			
Mean length of stay (days)	8.4	8.9	0.40
Mean age	84.0	85.0	0.16
Sex ratio			0.50
Male (%)	29	27	
Female (%)	71	73	
*Order characteristics*			
Mean number of medications/day/patient	12.1	12.3	0.87
Mean number of dosages/day/patient	23.0	23.6	0.83
Dosage form			0.67
Solid oral forms (%)	62.1	59.9	
Injections (%)	15.4	15.3	
Liquid oral forms (%)	12.5	10.4	
Oral powders (%)	4.8	6.5	
Inhalation forms (%)	1.7	3.9	
Percutaneous (%)	1.7	1.6	
Eye drops (%)	1.1	1.3	
Cream/ointments (%)	0.6	1.0	
*Nurse attendant characteristics*			
Experience (years)	*n*	*n*	1.00
0–1	3	4	
1–5	13	11	
>5	12	16	
*Unit characteristics*			
Unit occupancy rate (%)	97.3	96.6	

UDDS, unit dose dispensing system; WSS, ward stock system.

**Table 2:** Observed medication round characteristics for each period

	WSS	UDDS			
Period	Total	Total	*P*	UDDS1	UDDS2
Accompanied rounds	28	31		15	16
Number of patients	148	166		78	88
*Daytime distribution*			0.92		
Morning	247	303		102	201
Noon	112	170		73	97
Evening	256	310		203	107
Total	615	783		378	405
Mean number of medications per round	20.67	25.77	0.07	28.21	23.63
Mean administration time (minutes)	41.67	61.17	<0.05	64.64	58.13
Mean number of medications per patient per round	3.40	2.97	0.17	3.55	3.38
Mean nursing time per patient (minute/dose)	2.26	2.54	0.44	2.67	2.43

UDDS, unit dose dispensing system; WSS, ward stock system.

**Figure 1:**
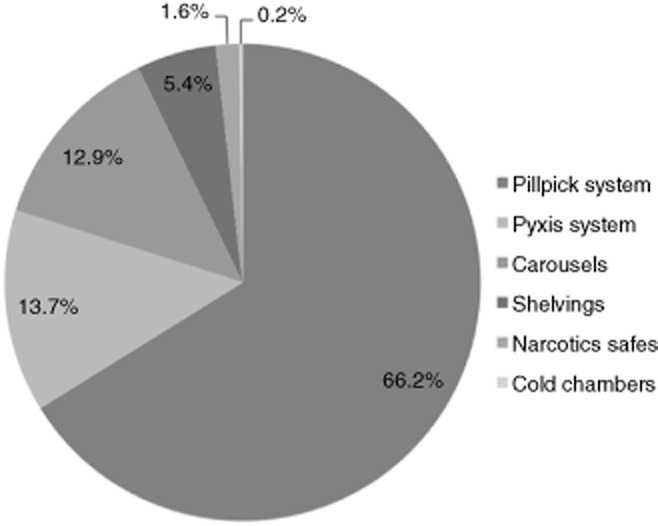
Distribution of doses dispensed through the different systems or storage units during the unit dose dispensing system period.

Systems or storage units used to dispense medications during the UDDS period are presented in Figure 1. A total of 79.9% of medications were dispensed using the Pillpick or Pyxis systems. For daily delivery, without taking into account the Pyxis system, 76.7% of dispensations were managed with the Pillpick system, and 14.9% with automated storage.

### MAE rates

A total of 1398 OEs were observed during the study, 615 during the WSS period and 783 during the UDDS period.

**Figure 2:**
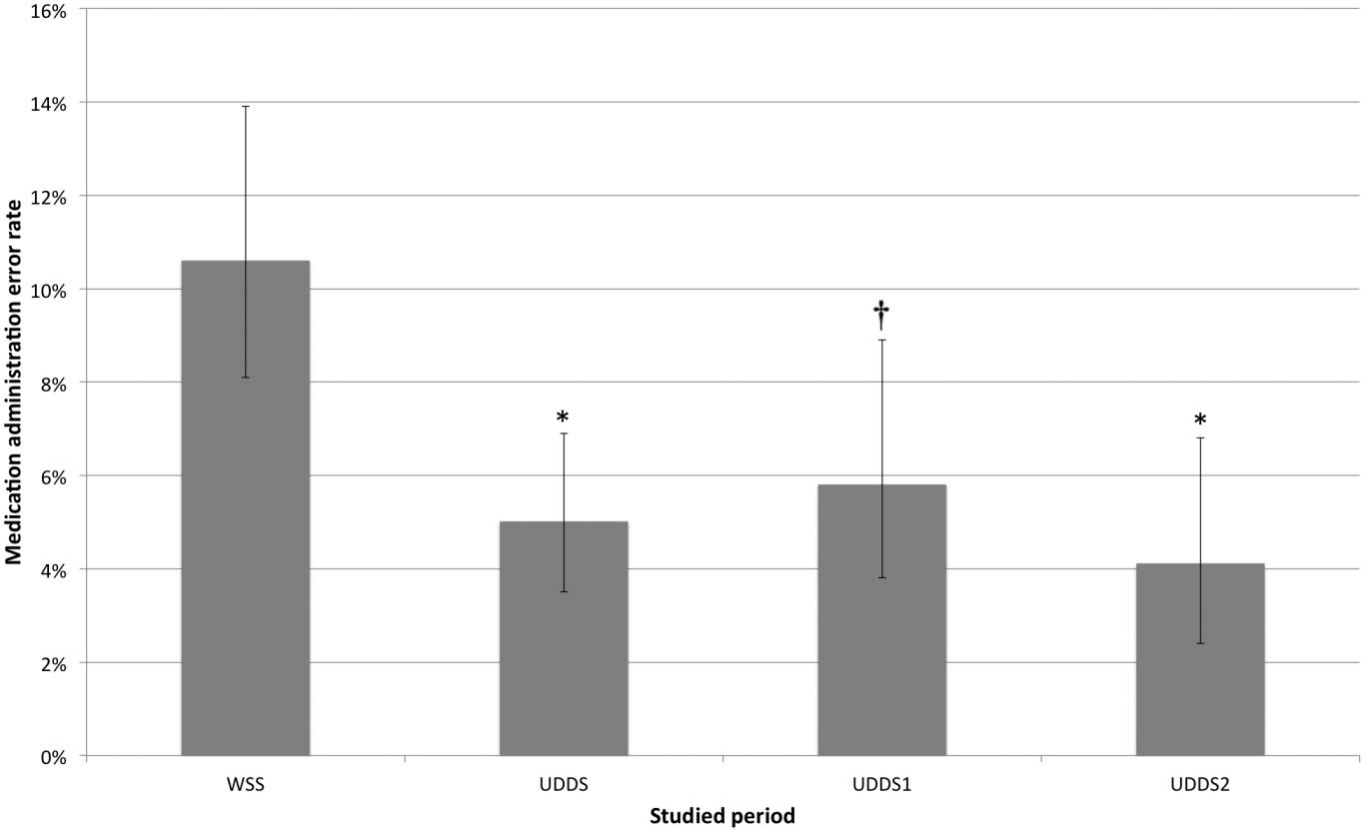
Medication administration error rates for each period. WSS, ward stock system; UDDS, unit dose dispensing system; UDDS1, unit dose dispensing system without electronic medication administration record; UDDS2, unit dose dispensing system with electronic medication administration record. Significant differences when compared to WSS are indicated by *P < 0.001 or †*P* < 0.01.

Observers did not halt administration because of any potential harmful error. The estimated rate of MAE is presented in Figure 2. Forty-one MAEs were disclosed (MAE rate 5.0%; 95%CI 3.5–6.9%) during the UDDS period in comparison to 74 MAEs (MAE rate 10.6%; 95%CI 8.1–13.9%) in the WSS period (*P* < 0.001, absolute reduction of 5.7%, relative reduction of 53%). For the UDDS1 and UDDS2 periods, 25 and 16 MAEs were observed, respectively (MAE rate 5.8%, 95%CI 3.8–8.9% in UDDS1; MAE rate 4.1%, 95%CI 2.4–6.8% in UDDS2). In comparison to the WSS period, the MAE rate observed in UDDS1 and UDDS2 periods decreased significantly by 45 and 62%, respectively (*P* = 0.02 and *P* = 0.001, respectively). Comparison of the UDDS1 and UDDS2 periods showed no significant difference (*P* = 0.30).

If administration is to be considered correct only if the medication is recognizable at the time of administration, that is, when its primary packaging is intact and still displays drug name and dosage, the WSS administration error rate reached 49.0% (95%CI 43–54%), while UDDS rates remained unchanged as every medication was packaged into single doses or labelled by the pharmacy; the corresponding relative reduction in administration error was 89.9%.

During the WSS period, at least one MAE occurred among 30.4% of the patients (45 patients) compared to a 19.9% rate (32 patients) during the UDDS period. This reduction was statistically significant (*P* < 0.05), with an absolute risk reduction of 10.5% and a relative risk reduction of 34.6%. One of every 10 patients who were switched from WSS to UDDS avoided a ME, or for every 15 medications administered a ME was prevented.

### MAE types

**Figure 3:**
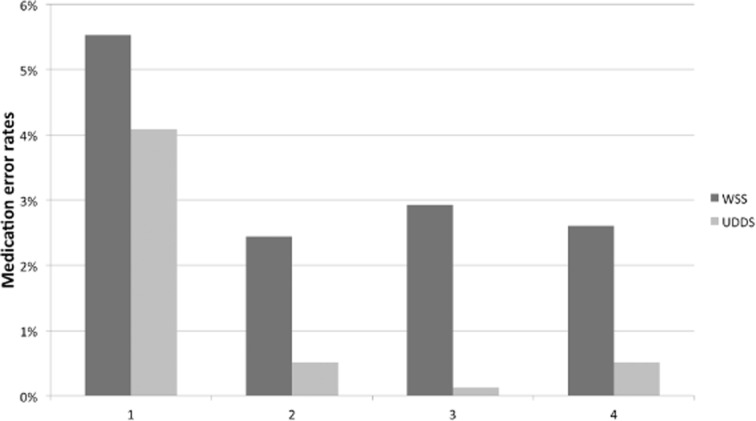
Medication administration error rates analysed according to type: type 1, omission; type 2, wrong dose; type 3, wrong drug; type 4, wrong time of administration.

Types of MAEs encountered are presented in Figure 3. Error types that decreased most during the UDDS period compared to the WSS one were wrong dosage (15 versus 4), wrong drug (18 versus 1) and wrong time of administration (7 versus 4). Non-administered drugs were observed in similar numbers between the two periods (34 versus 32). No extra dose was observed. Wrong dosage forms were observed once during the WSS period and once during the UDDS period, and have been included in the wrong drug category.

### Clinical error gravity assessment

Error gravity was statistically different (*P* < 0.01) between the two periods (Table 3), with a lower prevalence of type 2 and 3 errors (2: monitoring; 3: need for therapy or intervention) during the UDDS period.

**Table 3:** Medication error gravity for periods studied

Period	WSS (%)	UDDS (%)
0: No harm	21.1	23.2
1: Minimum harm with no monitoring expected	31.7	32.7
2: Monitoring	35.0	33.3
3: Need for therapy or intervention	12.2	10.7

UDDS, unit dose dispensing system; WSS, ward stock system.

## Discussion

The implementation of a UDDS was reduced by more than half of the discrepancies between medications ordered by the physician and medications actually administered to the patients by the nurses. Furthermore, drugs that were not identified at the patient's bedside were certified correct only after comparison with those in their original packaging, a check that is usually not performed by nurses. When we defined an unrecognizable medication as incorrect, the MAE rate in the WSS reached 49.0% and the error reduction rate after implementing a UDDS reached 89.9%. The number of patients subjected to one or more MAEs also significantly decreased during the UDDS period, with a relative risk reduction of 34.6%.

To our knowledge, this research is the first before-and-after observational study investigating an association between last-generation unit dose dispensing robots and AMDCs. However, other comparable studies have been carried out. The error rates observed in our study are slightly superior to data available in the literature. For example, Taxis *et al*. [9] reported an 8.0% error rate in a WSS and a 2.4% error rate for a UDDS. In our study, omission appeared to be the most frequent error when using a UDDS, but this may be correlated with the number of drugs included in the formulary and the compliance of physicians with prescribing drugs in the formulary. For non-formulary orders, pharmacists propose an equivalent drug available in the pharmacy but wait for the physician's approval before dispensing the drug. This latency may result in omission as the nurse may not have any of the prescribed drugs available in the unit. In a nursing home setting, Van den Bemt *et al*. [17] reported a much higher error frequency of 21%, but included administration technique errors such as incorrect crushing of tablets for intake with fluids or incorrect time of intake in relation to meals. The purpose of our study was to assess the discrepancy between the orders placed by the physician and what was really administered by the nurse. As physicians seldom specify a specific time of administration or may forbid the crushing of specific tablets, these were not classified as errors in our study.

All error types were reduced after changing the drug distribution system: incorrect dosage and incorrect drug were reduced by 79.1 and 93.7%, respectively. Omission was the most frequently observed ME during both periods and remained similar during the two periods. During the WSS period, drugs were sometimes not administered either because of temporary unavailability in the ward stock or because a brand name was ordered that differed from the one available in the hospital formulary. Non-formulary prescriptions were managed differently during the UDDS, as a pharmacist checked each order every day and could propose an equivalent formulary drug, or straightaway deliver an equivalent if a similar drug (same product and same form) was available. However, drugs were sometimes not administered during the UDDS period because a new order had been placed after the pharmacy had prepared the therapies. In such a case, the instruction was to adapt the therapy according to the AMDCs, or by reaching the 24/7 pharmacy. However, these instructions were not always followed. During the study, the robot was used 5 days a week and admissions were processed up to 1600 h. The robot is now working 6 days a week and up to 1800 h each day, so there should be fewer drug omissions. In our study, we considered a non-administered drug as an error as long as the physician did not clearly express ‘*pro re nata*’ conditions, but in certain situations, nurses' clinical judgement could result in medication doses being cancelled even if no ‘*pro re nata*’ condition was clearly expressed by the physician, and this may be included in the reported omissions. This may also explain why error gravity appeared to be different before and after implementing the UDDS, with a lower prevalence of the most serious errors. In our study, if nurses did not apply the physician's orders exactly as specified after assessment of the patient's condition, it was considered as an error, but the physician may not have considered that error to be serious. There may have been a lesser reduction in these kinds of ‘errors’ after changing the drug distribution system, but at the same time a greater reduction in real unintended errors, which may explain the observed modification in error gravity distribution. As we expected most MAEs to be minor, we did not use methods that were less sensitive to minor errors like the National Coordinating Council-Medication Error Reporting and Prevention [18]; otherwise, this result may not have been significant.

Errors were still observed during the UDDS period. The robot does not handle certain medications, such as those kept in large bottles or cold storage. These drugs are labelled by the pharmacy technician and delivered beside the rings, directly in the drawer or in a cold-maintaining package. It was still up to the nurse to prepare the right dose if bottles were dispensed. Technicians could still make a mistake labelling the packages, and nurses could prepare an incorrect dose (e.g. a 5 mL spoon instead of a 20 mL spoon).

No difference was observed when comparing the two CPOE systems. This may be attributable to an insufficient number of observations. However, the difference between the two systems may be more significant prior to prescription because of decision support systems (for example), and may have not been detected in our study.

This research has several limitations. We were not able to include intravenous or inhaled drugs in this study, because these drugs are administered separately from the main drug administration rounds. Moreover, less frequent errors may have escaped observation, for example, patient medication reversal in a two-bedroom. This kind of error cannot be prevented by unit dose delivering if not associated with a bar code reading system at the patient's bedside. However, this technology can be part of the automated UDDS, as all drugs are either single-dose packaged or labelled and can display a bar code, unlike the WSS. A MAE decrease may be related to factors other than a drug distribution system change only: CPOE and order checking by the pharmacy. CPOE eliminates rewriting by the nurse, even if no rewriting error was disclosed during our study, and ensures prescription readability. However, if a nurse has a problem reading an order, she usually asks the physician to confirm. The most significant role of these two elements is prior to placing an order, thanks to decision support systems or pharmacy advice. The lack of a control unit also limits the significance of our study. Even if no major change occurs in the geriatric department between WSS and UDDS periods apart from the drug distribution systems, it is impossible to assert that ward organization is absolutely equivalent between periods. The method used in this study has other inherent limitations: the effect of the observer on the observed nurse. But this effect is supposed to limit errors and would be expected to be similar in both periods, before and after the study.

## Conclusion

A drug distribution system including a UDDS and AMDCs could reduce MAEs when compared to the traditional WSS. However, the root causes of MAEs are not limited to drug distribution systems, as there can be discrepancies between the medication the physician intended to prescribe and the order placed in a CPOE, and even between the prescribed medication and the recommended drug according to evidence-based guidelines. In order to prevent MEs and further optimize patient medication management, a large spectrum of complementary solutions will be necessary. Assessments will be required to improve drug distribution systems with and without these complementary solutions. Economic assessments should also be carried out to compare ward and pharmacy staff workloads, medication consumption, and the cost of MAEs.
